# The protein phosphatase activity of PTEN is essential for regulating neural stem cell differentiation

**DOI:** 10.1186/s13041-015-0114-1

**Published:** 2015-04-18

**Authors:** Jingwen Lyu, Xiuya Yu, Lingjie He, Tianlin Cheng, Jingjing Zhou, Cheng Cheng, Zhifang Chen, Guoqiang Cheng, Zilong Qiu, Wenhao Zhou

**Affiliations:** Departments of Neonatology, Children’s Hospital of Fudan University, 399 Wanyuan Road, Shanghai, 201102 China; Institute of Neuroscience, Shanghai Institute of Biological Sciences, Chinese Academy of Sciences, Shanghai, 200031 China

**Keywords:** PTEN, Protein phosphatase activity, Neural stem cells, Differentiation, Neurogenesis

## Abstract

**Background:**

The tumor suppressor gene Phosphatase and tensin homolog (*PTEN*) is highly expressed in neural progenitor cells (NPCs) and plays an important role in development of the central nervous system. As a dual-specificity phosphatase, the loss of PTEN phosphatase activity has been linked to various diseases.

**Results:**

Here we report that the protein phosphatase activity of *Pten* is critical for regulating differentiation of neural progenitor cells. First we found that deletion of *Pten* promotes neuronal differentiation. To determine whether the protein or lipid phosphatase activity is required for regulating neuronal differentiation, we generated phosphatase domain-specific *Pten* mutations. Interestingly, only expression of protein phosphatase-deficient mutant Y138L could mimic the effect of knocking down *Pten*, suggesting the protein phosphatase of *Pten* is critical for regulating NPC differentiation. Importantly, we showed that the wild-type and lipid phosphatase mutant (G129E) forms of *Pten* are able to rescue neuronal differentiation in *Pten* knockout NPCs, but mutants containing protein phosphatase mutant cannot. We further found that *Pten*-dependent dephosphorylation of CREB is critical for neuronal differentiation.

**Conclusion:**

Our data indicate that the protein phosphatase activity of PTEN is critical for regulating differentiation of NSCs during cortical development.

**Electronic supplementary material:**

The online version of this article (doi:10.1186/s13041-015-0114-1) contains supplementary material, which is available to authorized users.

## Background

Embryonic neural stem cells (NSCs) self-renew and also differentiate into neurons, astrocytes, and oligodendrocytes [1]; maintaining a balance between these two processes is essential for normal brain development [2,3], and requires the coordinate regulation of multiple signaling pathways [4].

*Phosphatase and tensin homolog deleted on chromosome 10* (*PTEN*) is a tumor suppressor and autism-related gene that regulates proliferation and differentiation in a variety of cell types [5-8]. As a dual-specificity phosphatase, the PTEN protein contains catalytic and C2 domains and a C-terminal PDZ-binding motif [9]. The lipid phosphatase activity is required for antagonizing the phosphatidylinositol 3-kinase (PI3K)/AKT pathway, which controls cell proliferation and survival [10-13]; the major PTEN substrates are cyclic AMP response element-binding protein [14] and PTEN itself [15].

The complete inactivation of PTEN can lead to seizures, highlighting its importance in normal brain function. The G129E mutation, observed in Cowden syndrome, specifically abolishes the lipid phosphatase activity [16], while the Y138L mutation results in the loss of protein phosphatase function [17]. Embryonic *Pten* null mice have enlarged brains [7,8,18], while *Pten* deletion in adult mice enhanced NSC proliferation and differentiation into neurons, suppressed cell death, and resulted in an increased cell size [5-8]. Despite these observations, the *Pten* deletion mouse model has not provided a clear insight into the role or mechanism of PTEN function in NSCs during embryonic development.

The present study investigated whether the catalytic activity of PTEN is involved in NSC differentiation in embryos. Using NSC-derived neurosphere cultures transduced with lentivirus expressing mutant protein, it was found that the protein phosphatase activity is specifically required for balancing NSCs fate determination.

## Results

### Pten-deficient NSCs differentiate precociously into neurons

**Figure 1:**
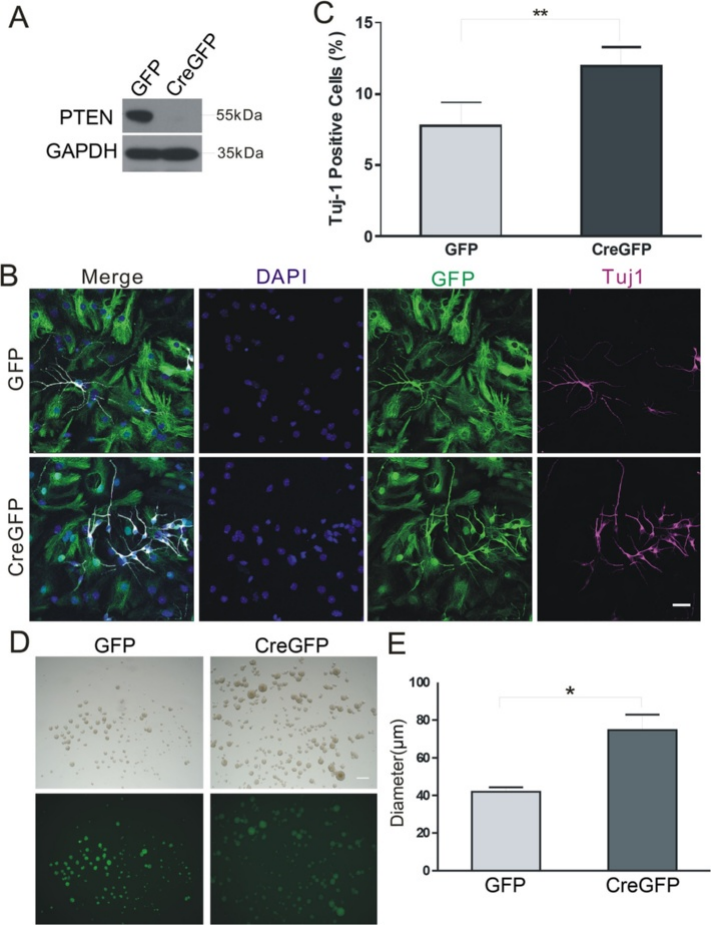
*Pten* deletion promotes differentiation of NSCs into neurons and enlarges neurosphere. **(A)** Neurospheres generated from *Pten* ^*loxp/loxp*^ NSCs, were transfected with lentivirus expressing GFP (control) or Cre-GFP (for *Pten* deletion), and induced to differentiate by growth factor withdrawal. PTEN protein expression was abolished by Cre-GFP-mediated *Pten* removal, as detected by western blotting. GAPDH was used as a loading control. **(B)** The fate change of differentiated NSCs was ascertained by labeling with an antibody against the neuronal marker Tuj1. GFP^+^ cells represent the cells transfected with lentivirus. Nuclei were stained with DAPI. Scale bar = 40 μm. **(C)** A greater number of Tuj1^+^GFP^+^ cells was observed upon *Pten* deletion compared to *Pten* ^*loxP/loxP*^ controls. Data are expressed as mean ± SD. **P < 0.01 (n > 800 cells per group from each experiment). **(D)** Neurospheres cultured 3 days after harvesting through flow cytometry in proliferation condition culture. Scale bar = 100 μm. **(E)** Values are expressed as the neurospheres diameter. Data are expressed as mean ± SD. *P < 0.05 (The average sizes with standard deviations determined for all neurospheres in one well).

To evaluate the function of *Pten* in embryonic NSCs, the fate of neurospheres derived from *Pten*^*loxP/loxP*^ embryos at E12.5 and maintained under proliferating culture conditions was examined. We first isolated neural stem cells from mouse brain at E12.5 and stained with neural progenitor marker Nestin and found that majority of neural stem cells were Nestin positive cells (Additional file 1: Figure S1A). After five days, neurospheres were infected with lentivirus expressing GFP (control) or Cre-GFP (for *Pten* deletion), and cells were induced to differentiate as described in section 2.2. After 4–5 days, there was no PTEN protein expressed by NSCs transduced with Cre-GFP (Figure 1A). Cells were examined the expression of the differentiated neuron marker Tuj1 (Figure 1B). The number of Tuj1^+^GFP^+^ cells was 53.2% higher in PTEN-deficient cells than controls (11.9 ± 1.4 vs. 7.8 ± 1.6; P < 0.01) (Figure 1C).

We further examined whether deletion of *Pten* may affect the proliferation of neural stem cells. Indeed, we found that deletion of *Pten* leads to a significant increase of diameter of neurospheres, comparing to GFP only virus infections (Figure 1D, E). We also stained neural stem cells with Caspase3 and found that there was not significant neuronal cell death when *Pten* was knockout (Additional file 1: Figure S1B). To address whether *Pten* deletion would affect glial differentiation, we examined the glia cells by staining GFAP in NSCs under differentiation condition with or without *Pten* deletion. We found that *Pten* deletion did not affect GFAP-positive cell numbers (Additional file 2: Figure S2A, B). We speculate that in our preparation and culture conditions, NSCs preferred to undergo neuronal differentiation rather than glial differentiation. Neither we found that PTEN lipid and protein phosphatase activity show effect on the glial differentiation (Additional file 3: Figure S3).

To determine whether PTEN-deficient neurons are able to develop normally, we stained neuronal marker MAP-2 and NeuN on the neurons cultured over 14–17 days after neuronal differentiation and found that PTEN-deficient neurons were still able to differentiate into mature neurons (Additional file 4: Figure S4A, B). Interestingly, we found that the total neurite length is significantly increased when *Pten* was deleted, in consistent with previous finding that *Pten* is critical for neuronal dendritic growth (Additional file 5: Figure S5A,B). Thus we think that the increase of neurite growth after *Pten* deletion in our experiments echo this previous finding that loss of *Pten* lead to increased dendritic length by regulating Akt/mTOR/S6K pathway[18].

### PTEN protein phosphatase activity maintains NSCs in a progenitor state

**Figure 2:**
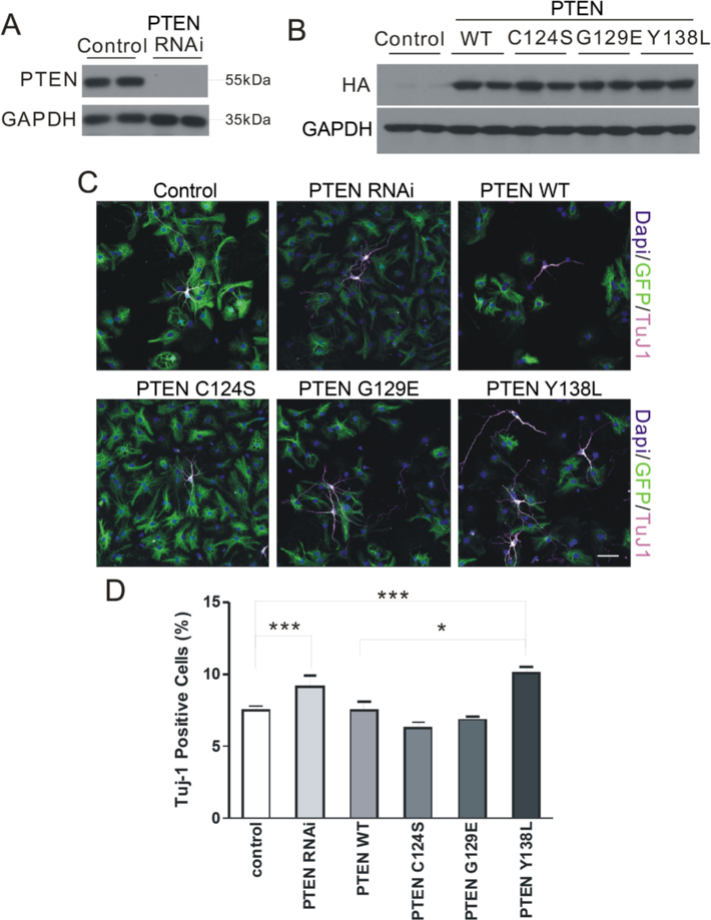
PTEN protein phosphatase activity maintains NSCs in a progenitor state. **(A)** Cells were transduced with WT or mutant PTEN (Y138L, G129E, or C124S: mutations in either protein or lipid phosphatase domain, or both, respectively) or PTEN shRNA. Protein expression was absent in PTEN RNAi cells. **(B)** Lentiviral transduction of WT, Y138L, G129E, and C124S resulted in the expression of a 55 kDa band corresponding to the PTEN protein, as seen by western blotting. GAPDH was used as a loading control. **(C)** NSCs transduced with GFP, PTEN shRNA, or WT, Y138L, G129E, or C124S constructs were labeled with an antibody against the neuronal marker Tuj1. Scale bar = 40 μm. **(D)** Increased neuronal differentiation was observed upon loss of PTEN protein phosphatase activity (Y138L). Data are expressed as mean ± SD. *P < 0.05, ***P < 0.001 (n > 800 cells per group from each experiment).

To evaluate the mechanism underlying the observed effects of PTEN in NSC differentiation, WT or mutant PTEN—harboring a loss-of-function mutation in either the protein (Y138L) or lipid (G129E) phosphatase domain, or both (C124S)—or PTEN shRNA was overexpressed in NSCs. PTEN protein expression was absent in PTEN RNAi cells (Figure 2A). A 55 kDa band was observed upon lentiviral transduction of NSCs with WT, Y138L, G129E, and C124S, corresponding to the PTEN protein (Figure 2B). NSCs were infected with lentivirus expressing GFP, PTEN shRNA, or WT, Y138L, G129E, or C124S (Figure 2C). There was no difference in the number of Tuj1^+^ neurons upon infection of cells with GFP control, WT, G129E, or C124S constructs. However, an increase in neuronal production was observed upon overexpression of Y138L (10.0 ± 0.9 vs. 7.48 ± 0.6 in GFP controls; P < 0.001), 34.2% more than for PTEN WT overexpression (10.0 ± 0.9 vs. 7.48 ± 1.1; P < 0.05) (Figure 2D).

**Figure 3:**
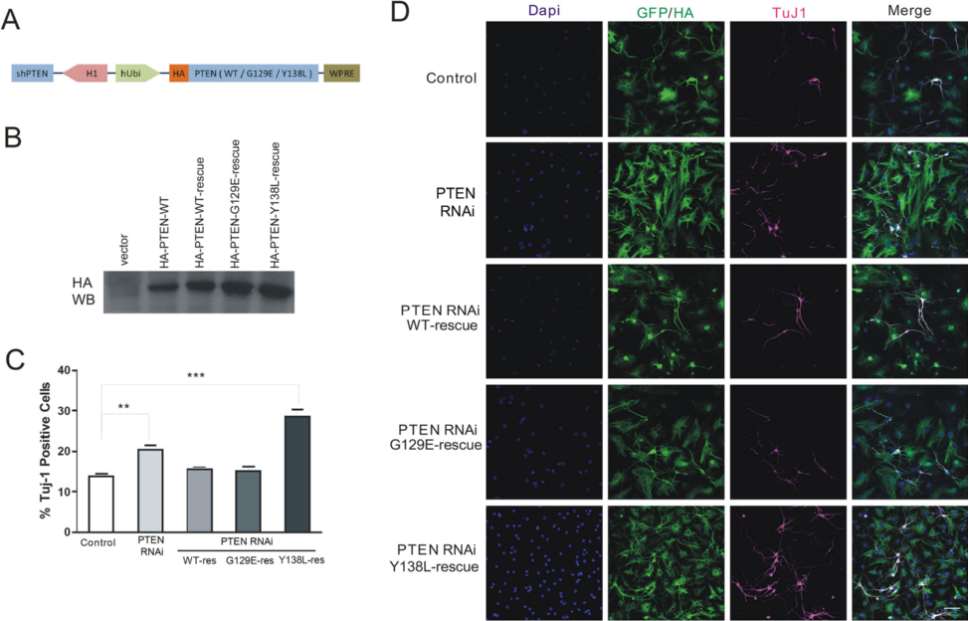
RNAi-resistant PTEN rescues the effect of PTEN knockdown. **(A)** Schematic illustration of lentiviral-based PTEN wild type, G129E, Y138L rescue constructs. **(B)** 293 T cells were transfected with vector, HA-PTEN-WT, HA-PTEN-WT-rescue, HA-PTEN-G129E-rescue, HA-PTEN-Y138L-rescue resulted in the expression of a 55 kDa band corresponding to the PTEN protein, as seen by western blotting. **(C)** The data show the Tuj1 positive cells in control, PTEN RNAi, PTEN RNAi + HA-PTEN-(WT, G129E, Y138L)-rescue conditions respectively. **(D)** NSCs transduced with GFP, PTEN RNAi, or PTEN RNAi + HA-PTEN-(WT, G129E, Y138L)-rescue constructs were labeled with an antibody against the neuronal marker Tuj1. Scale bar = 40 μm. Data are expressed as mean ± SD. **P < 0.01, ***P < 0.001 (n > 800 cells per group from each experiment).

To further confirm this result, we made RNAi-resistant rescue constructs of wild-type, G129E, and Y138L mutant of PTEN (Figure 3A, B). In experiments described above, we found that shRNA-resistant HA-PTEN WT, shRNA-resistant HA-PTEN G129E rescued the effect of PTEN shRNA (Figure 3A-D). Surprisingly, an increase in neuronal production was still observed upon shRNA-resistant HA-PTEN Y138L (Figure 3C, D). The results of this experiment provide further evidence that PTEN protein phosphatase is critical for neuronal differentiation in embryonic NSCs.

### Expression of WT PTEN rescues the precocious differentiation induced by inactivation of PTEN protein phosphatase function

**Figure 4:**
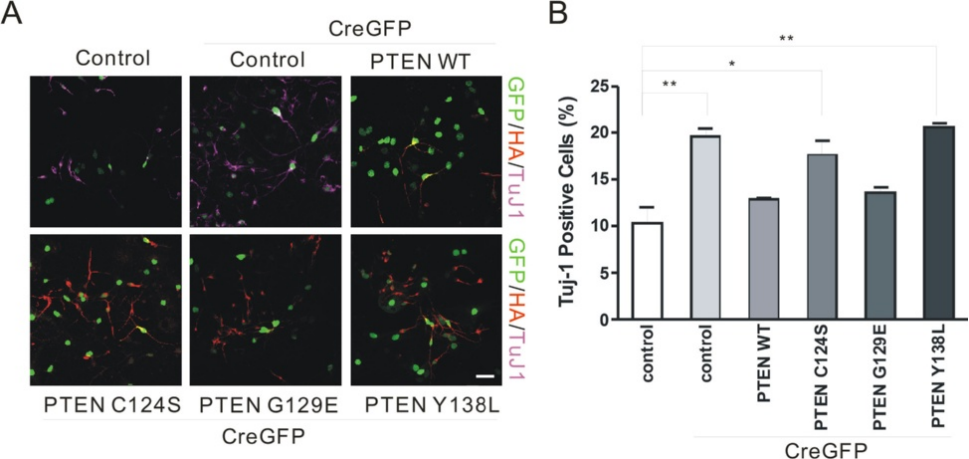
Expression of wild-type, but not the protein phosphatase mutant form of PTEN, rescues the accelerated differentiation induced by the genetic deletion of PTEN. **(A)** *Pten* ^*loxP/loxP*^ NSCs were co-transduced with Cre-GFP and either GFP, or HA-tagged WT PTEN, Y138L, G129E, or C124S constructs; co-expression of the two constructs was visualized by simultaneously labeling cells with antibodies against GFP and HA. The mutations are described in the Figure 2 legend. Scale bar = 40 μm. **(B)** Overexpression of WT PTEN abolished the increase in the number of Tuj1^+^ cells induced by the loss of PTEN (Cre-GFP + HA PTEN-WT). G129E, which retains protein phosphatase activity, also rescued the precocious differentiation. Data are expressed as mean ± SD. *P < 0.05, **P < 0.01 (n > 200 cells per group from each experiment).

To confirm that the protein phosphatase activity of PTEN mediates NSC differentiation, *Pten*^*loxP/loxP*^ NSCs were co-transduced with Cre-GFP and GFP, WT PTEN, Y138L, G129E, or C124S constructs (Figure 4A). Overexpression of WT PTEN abolished the increase in the number of Tuj1^+^ cells induced by the loss of PTEN (12.9 ± 0.3 vs. 10.3 ± 3.0 for GFP only; P = 0.13), while this was not observed when the GFP control lentivirus was co-infected with Cre-GFP (Figure 4B). G129E, which retains protein phosphatase activity, also rescued the excessive differentiation induced by PTEN deficiency (13.6 ± 1.1 Tuj1+ cells vs. 10.3 ± 3.0 for GFP only; P = 0.09).

Previous studies have shown that PTEN is required for the physiological function of NSCs, and PTEN ablation in the mouse cortex and hippocampus leads to macrocephaly, social behavior and learning deficits, seizures, and increased anxiety [5,8,18]. More recently, increased proliferation and differentiation rates have been observed in *Pten*-deficient adult NSCs, leading to the production of hypertrophic neurons and ultimately to the early depletion of the NSCs pool. In *Pten* mutant mice, NSCs in the hippocampus preferentially differentiated into astrocytes rather than neurons [6,7]. In accordance with effects observed in adult NSCs [7], the results of present study provide evidences for increased neuronal differentiation in embryonic NSCs upon loss of PTEN.

### PTEN-dependent CREB dephosphorylation is critical for neural stem cell differentiation

**Figure 5:**
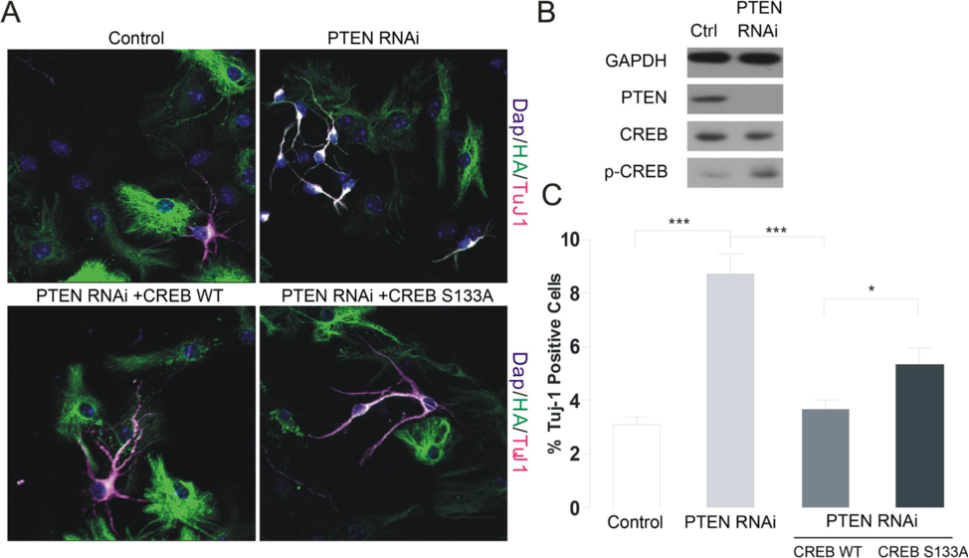
PTEN-dependent CREB dephosphorylation is critical for regulating neural stem cell differentiation. **(A)** NSCs transduced with GFP, PTEN RNAi, or PTEN RNAi + CREB (WT), PTEN RNAi + CREB (S133A) constructs were labeled with an antibody against the neuronal marker Tuj1. Scale bar = 40 μm. **(B)** Western analysis of CREB phosphorylation in control and PTEN RNAi primary cultured neurons. **(C)** Quantification of experiments in **(A)**. Data are expressed as mean ± SD. *P < 0.05 **P < 0.01, ***P < 0.001 (n > 800 cells per group from each experiment).

It is reported that PTEN dephosphorylated CREB directly through its protein phosphatase activity [14]. Indeed, we confirmed that the CREB phosphorylation significantly increased in PTEN-depleted neurons by RNAi, indicating PTEN also dephosphorylate CREB protein at S133 site in NSCs and mouse neurons (Figure 5B). Next we would like to examine whether CREB is required for PTEN-regulated neural differentiation. We overexpressed wild-type and phosphorylation mutant S133A form of CREB after knocking down PTEN (Figure 5A, C). Interestingly, we found that wild-type CREB, but not S133A mutant, could rescue the abnormal neuronal differentiation caused by PTEN deletion, suggesting that PTEN may regulate neuronal differentiation through dephosphorylating CREB at S133 site (Figure 5A, C).

## Conclusions

The loss of PTEN phosphatase activity has been linked to various diseases. For instance, PTEN G129E was identified in two families with Cowden disease [19]. In glioblastoma cells, PTEN lipid and protein phosphatase activities independently inhibit cell proliferation, and both are coordinately required to effectively inhibit cell invasion [17]. The protein but not the lipid phosphatase activity is involved in the decrease in dendritic spine density of neurons overexpressing wild-type PTEN [15]. Here it was shown that NSC maintenance is abolished when the protein phosphatase domain of PTEN is inactivated, leading to accelerated neuronal differentiation. This activity may be self-directed (i.e., via dephosphorylation of PTEN) [20], and is likely distinct from its role in the PI3K/AKT pathway, which is antagonized by PTEN lipid phosphatase activity and regulates cell proliferation, survival, and motility [12]. Moreover, several reports have highlighted the link between PTEN protein phosphatase activity and the regulation of cell migration and invasion [20].

In summary, the findings of this study demonstrate that PTEN deficiency promotes neurogenesis in embryonic NSCs, and the protein phosphatase activity of PTEN is specifically required to maintain NSCs in a self-renewing state during embryonic development. Additional experiments are required to determine if there are other substrates targeted by PTEN-mediated dephosphorylation in the differentiation of the NSC population during embryonic development.

## Materials and methods

### Mice

*Pten*^*loxP/loxP*^ mice were purchased from the Jackson Laboratory (Farmington, CT, USA). All experimental procedures were performed according to the guidelines and under the approval of the Animal Care and Use Committee of the Institute of Neuroscience of the Chinese Academy of Sciences.

### Neurosphere cultures and lentivirial transduction

NSCs were obtained from mice at embryonic day (E)12.5. Briefly, the cortex was dissected and placed in ice-cold phosphate-buffered saline (PBS; Invitrogen); the tissue was triturated, then passed through a 70-μm nylon cell strainer (BD Biosciences, Franklin Lakes, NJ, USA) to dissociate the cells, which were plated at 2 × 10^5^ cells/ml in Dulbecco’s Modified Eagle’s Medium (DMEM)/F12 medium with 2% B27 supplement without vitamin A, 1% N2 supplements, and 10 ng/ml each of recombinant human basic fibroblast growth factor (bFGF) and epidermal growth factor (EGF; all from Invitrogen, Carlsbad, CA, USA). Neurospheres were passaged every five days. To induce NSC differentiation, resuspended cells were plated in poly-l-ornithine-coated wells of an 8-well chambered slide (Nunc Lab-Tek II; Thermo Fisher Scientific, Waltham, MA, USA) at a density of 5 × 10^4^ cells/well in the same supplemented DMEM/F12 medium, but with 1% fetal bovine serum (Invitrogen) and without growth factors. Lentiviruses (10^8–9^ vg/ml) were tansduced in a single-cell suspension. Cells were allowed to differentiate for five days. For measuring the size of neurospheres, the NSCs were transfected with lentivirus expressing GFP or CreGFP at Day 1. At Day 3, GFP positive cells were collected by flow cytometry. The GFP positive cells were cultured at a density of 5000 cells/well in a 6-well plate. At day 6, the neurospheres were pictured and the sizes were measured.

### Immunocytochemistry

Cells were fixed in 4% paraformaldehyde in PBS. After washing in PBS, cells were blocked in blocking buffer (3% bovine serum albumin and 0.2% Triton X-100 in PBS), then incubated with primary and secondary antibodies diluted in blocking buffer. Cell nuclei were stained with DAPI. The following primary antibodies were used: goat α-green fluorescent protein (GFP; 1:500; Millipore, Billerica, MA, USA), rabbit α-Tuj1 (1:1,000; Invitrogen), goat α-HA (1:1,000; Millipore), and mouse α-GFP (1:2,000; Millipore), chicken α-GFAP (1:1000; Abcam, Cambridge, UK), mouse α-nestin (1:1000; R&D system), mouse α-MAP-2(1:500, Millipore), rabbit α-NeuN(1:1000, Miliipore).

### Western blotting

Total protein was extracted from NSCs cultured samples in RIPA buffer using a standard protocol. Samples were resolved by 8% SDS-PAGE and transferred to a polyvinylidene fluoride membrane (Millipore). Membranes were probed with the following primary antibodies: α-PTEN (1:1000; Cell Signaling Technology, Danvers, MA, USA), α-HA (1:2500; Abmart, Arlington, MA, USA), α-CREB (1:1000; Cell Signaling Technology, Danvers, MA, USA), α-p-CREB(S133) (1:1000; Cell Signaling Technology, Danvers, MA, USA) and α-glyceraldehyde 3-phosphate dehydrogenase (GAPDH; 1:5000; Abcam, Cambridge, UK), followed by incubation with a horseradish peroxidase-conjugated secondary antibody and processing with an enhanced chemiluminescence kit (Thermo Fisher Scientific).

### Plasmid construction

The RNAi targeting sequence for mouse *Pten* was 5′-AGA CAA GGC CAA CCG ATAC-3′. The wild-type (WT) *Pten* overexpression construct was generated by subcloning the ORF with an HA tag between the BamHI and EcoRI sites of the FUGW vector (Addgene). For the replacement construct, the *Pten* shRNA sequence was inserted into the PacI site of the expression vector under the control of H1 promoter. The following forward and reverse primer pairs were used to generate the PTEN mutation constructs: C124S, 5′-TCA TGT TGC AGC AAT TCA CTC T-3′ and 5′-CCG TCC CTT TCC AGC TTT-3′; G129E, 5′-ACT GTA AAG CTG GAA AGG AA-3′ and 5′-CAC AAA TCA TTA CAC CAG TCC G-3′; Y138L, 5′-CTC TTA TTG CAT CGG GGC AAA TTT TTA AA-3′ and 5′-AAT TTG CCC CGA TGC AAT AA-3′. The shRNA-resistant HA-PTEN was generated by introduction of six silent mutations into the sequence of *Pten* targeted by the shRNA, indicated in the following by lower case letters: 5′-GAt AAa GCt AAt aGg TAC-3′.

### Quantification

For counting percentage of differentiated cells, five images/sections were acquired randomly under a 10х object lens, immunolabeled cells were counted manually. Over 800 cells were counted per group from each experiment. For measurement of total neurite length, the images were obtained under a 40х object lens. The lengths of 30 neurons in each experimental group were measured from 4 independent experiments. All the data were measured using Image J.

### Statistical analysis

All results were expressed as mean ± SD and were analyzed using Stata v. 10 software (Stata Corporation, College Station, TX USA). A two-sample t test with equal variance was used to compare results from each group. A P value < 0.05 was considered statistically significant.

## Additional files

Additional file 1: Figure S1. Typical neurosphere and cell death detection. (A) Neurosphere after 5 days culture was shown by nestin staining (red). (B) Rarely cell death (Caspase 3 positive cells) was observed in differentiated cells after GFP or CreGFP lentivirus transfection. Additional file 2: Figure S2. PTEN deletion in NSCs has no effect on differentiation of glial cells. (A) The fate change of differentiated NSCs was ascertained by labeling with an antibody against the astrocyte marker GFAP. GFP^+^ cells represent the cells transfected with lentivirus. Nuclei were stained with DAPI. Scale bar = 40 μm. (B) No significant changes on GFAP^+^GFP^+^ cells was observed upon *Pten* deletion compared to *Pten* ^*loxP/loxP*^ controls. Data are expressed as mean ± SD. (n > 800 cells per group from each experiment). Additional file 3: Figure S3. PTEN lipid and protein phosphatase activity shows no effect on the glial differentiation of NSCs. (A) The fate change of differentiated NSCs was ascertained by labeling with an antibody against the astrocyte marker GFAP. GFP^+^ cells represent the cells transfected with lentivirus. Nuclei were stained with DAPI. Scale bar = 40 μm. (B) No significant changes on GFAP positive cells after each PTEN mutant overexpression were observed. Data are expressed as mean ± SD. (n > 800 cells per group from each experiment). Additional file 4: Figure S4. Neurons, differentiated from NSCs, develop into mature neurons. (A-B) Cells differentiated from NSCs 10 days after GFP or CreGFP lentivirus transfectionwere labeled with mature neuronal markers MAP2 and NeuN. Scale bar = 40 μm. Additional file 5: Figure S5. Total neurite length of control and *Pten* ^*−/−*^ neurons. (A) Representative image of neurite growth of differentiated neurons at 5 days after transfectionwith GFP or CreGFP lentivirus. Scale bar = 40 μm (B) The quantification plot of neurite length of differentiated neurons. Transfection with lentivirus expressing CreGFP showed a remarkable increase in neurite length compared to control.

## Abbreviations

bFGF: Basic fibroblast growth factor
EGF: Epidermal growth factor
GFP: Green fluorescent protein
NSCs: Neural stem cells
PI3K: Phophotidylinositol 3-kinase
PTEN: Phosphatase and tensin homolog deleted on chromosome 10
CREB: CAMP response element-binding protein
