# Supplementary figures and images for: The protein phosphatase activity of PTEN is essential for regulating neural stem cell differentiation
Source: Mol Brain. 2015 Apr 18;8:26. doi: 10.1186/s13041-015-0114-1 (PMC4427940; doi:10.1186/s13041-015-0114-1)

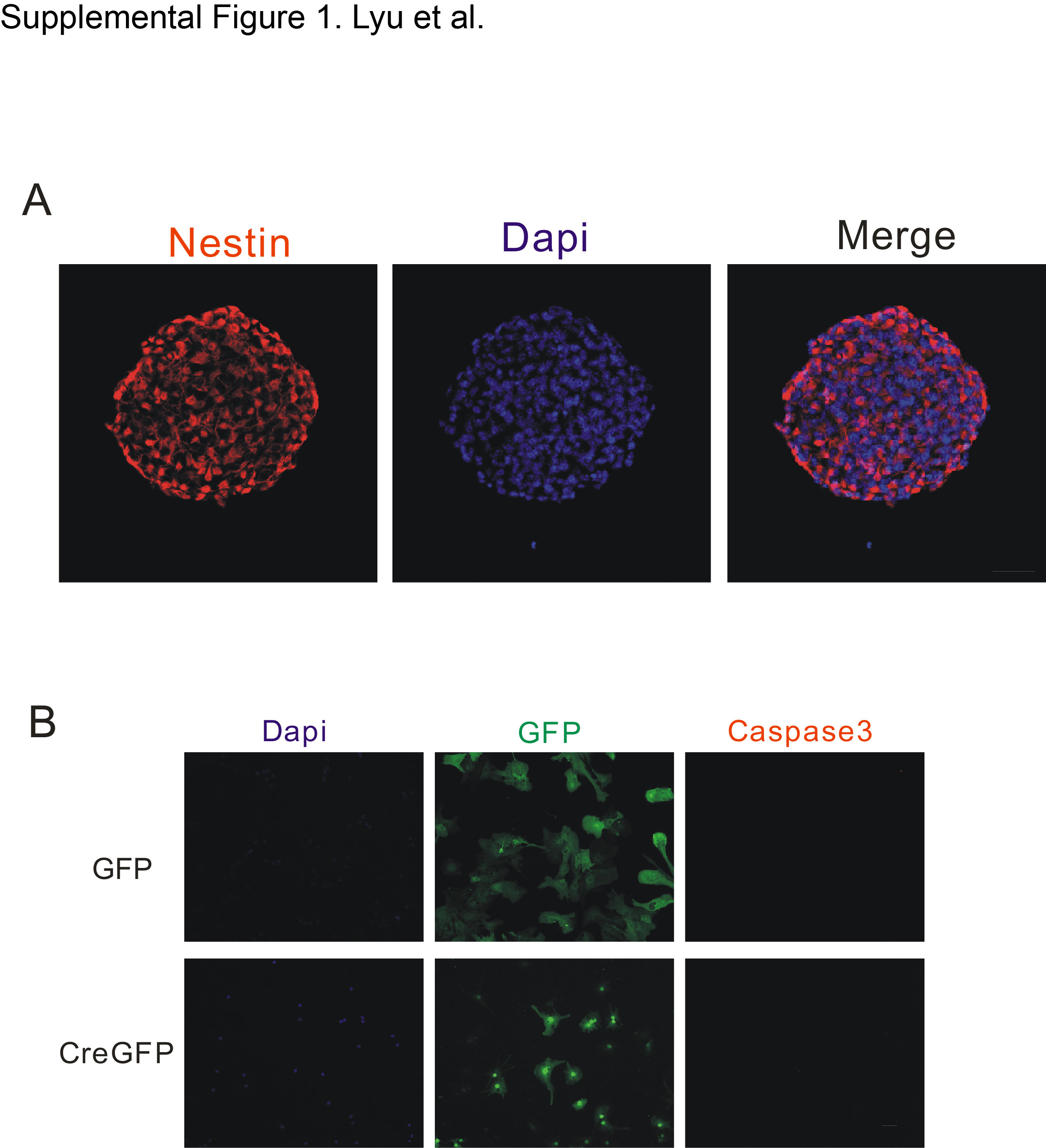

Supplement: Additional file 1: Figure S1. — Typical neurosphere and cell death detection. (A) Neurosphere after 5 days culture was shown by nestin staining (red). (B) Rarely cell death (Caspase 3 positive cells) was observed in differentiated cells after GFP or CreGFP lentivirus transfection. [file 13041_2015_114_MOESM1_ESM.jpeg]

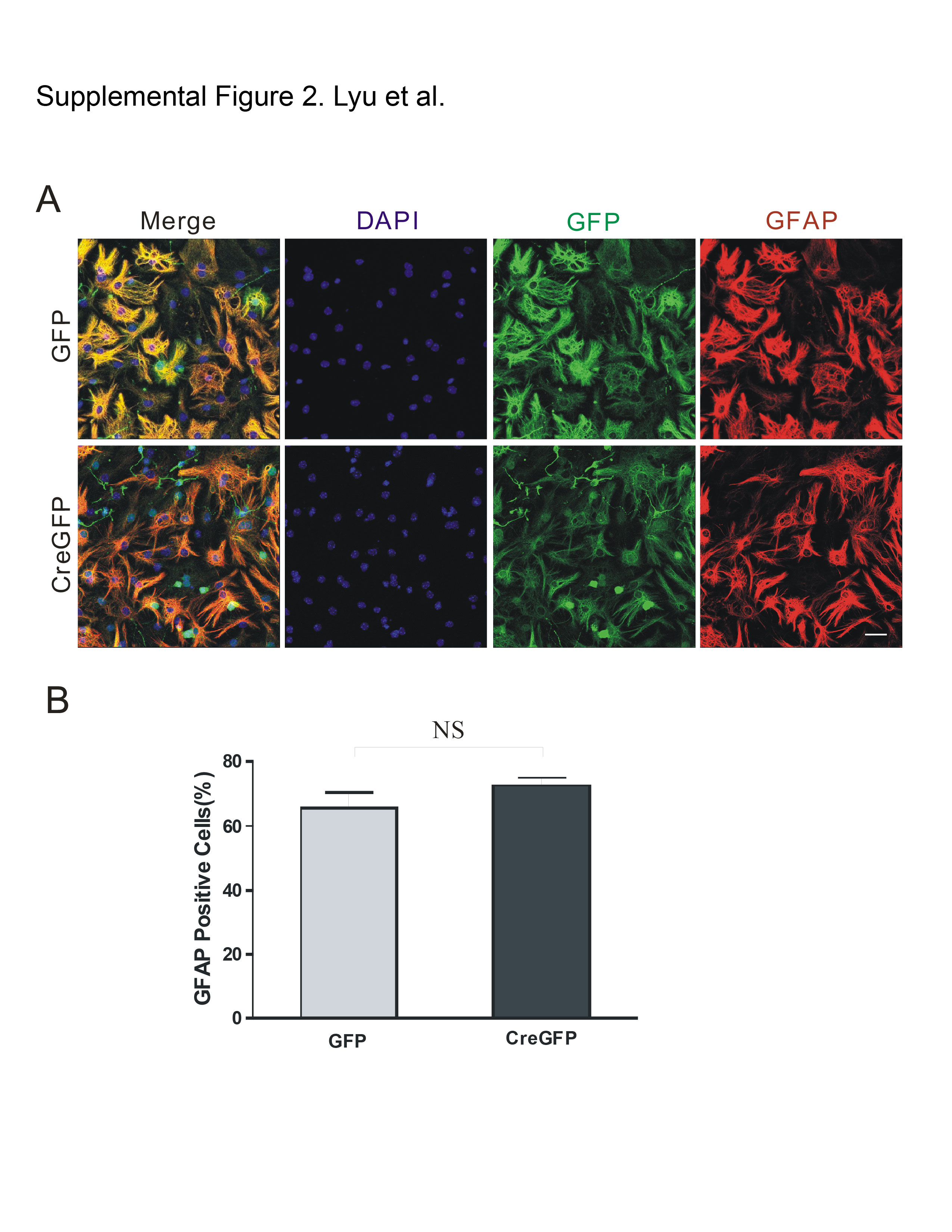

Supplement: Additional file 2: Figure S2. — PTEN deletion in NSCs has no effect on differentiation of glial cells. (A) The fate change of differentiated NSCs was ascertained by labeling with an antibody against the astrocyte marker GFAP. GFP+ cells represent the cells transfected with lentivirus. Nuclei were stained with DAPI. Scale bar = 40 μm. (B) No significant changes on GFAP+GFP+ cells was observed upon Pten deletion compared to Pten loxP/loxP controls. Data are expressed as mean ± SD. (n > 800 cells per group from each experiment). [file 13041_2015_114_MOESM2_ESM.jpeg]

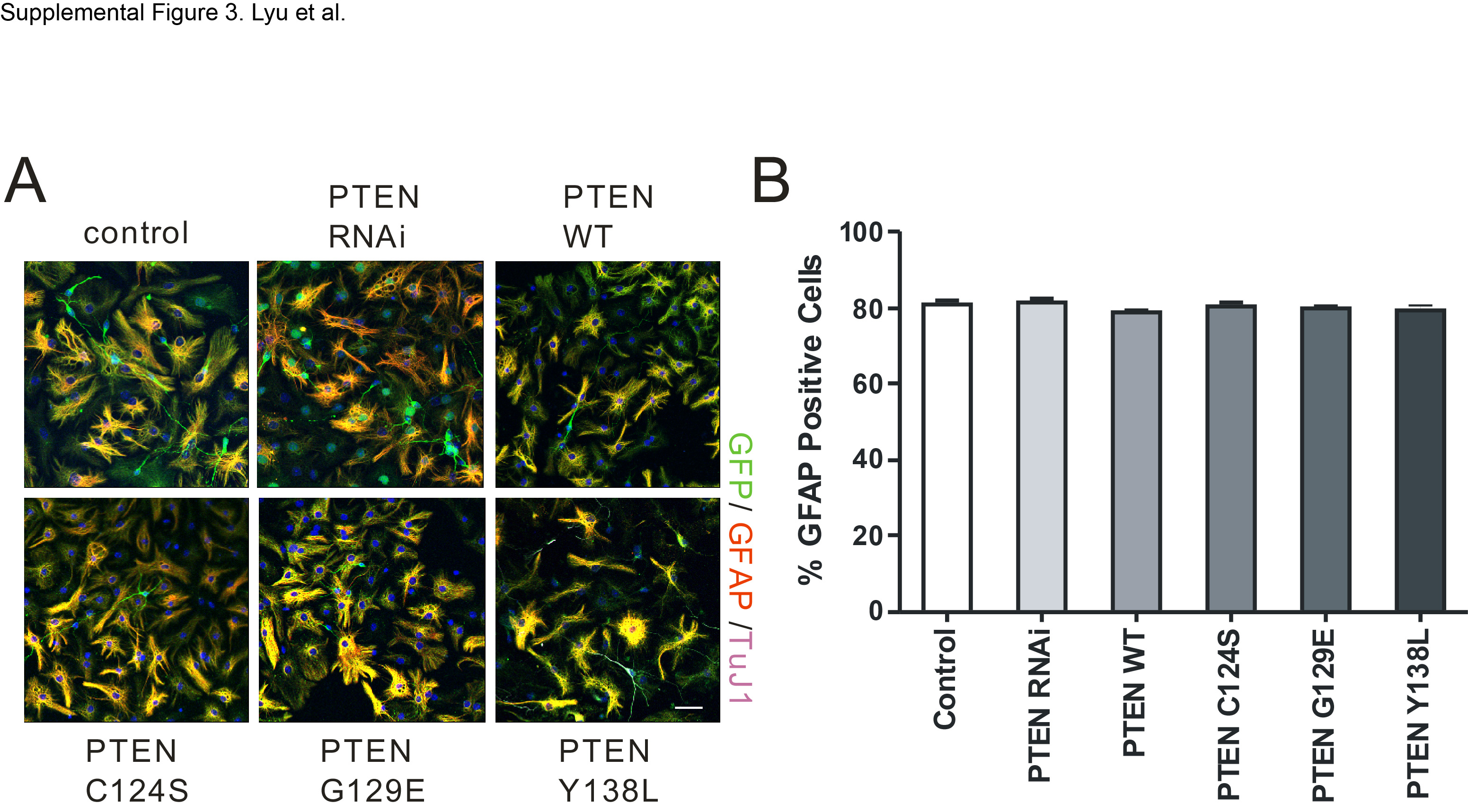

Supplement: Additional file 3: Figure S3. — PTEN lipid and protein phosphatase activity shows no effect on the glial differentiation of NSCs. (A) The fate change of differentiated NSCs was ascertained by labeling with an antibody against the astrocyte marker GFAP. GFP+ cells represent the cells transfected with lentivirus. Nuclei were stained with DAPI. Scale bar = 40 μm. (B) No significant changes on GFAP positive cells after each PTEN mutant overexpression were observed. Data are expressed as mean ± SD. (n > 800 cells per group from each experiment). [file 13041_2015_114_MOESM3_ESM.jpeg]

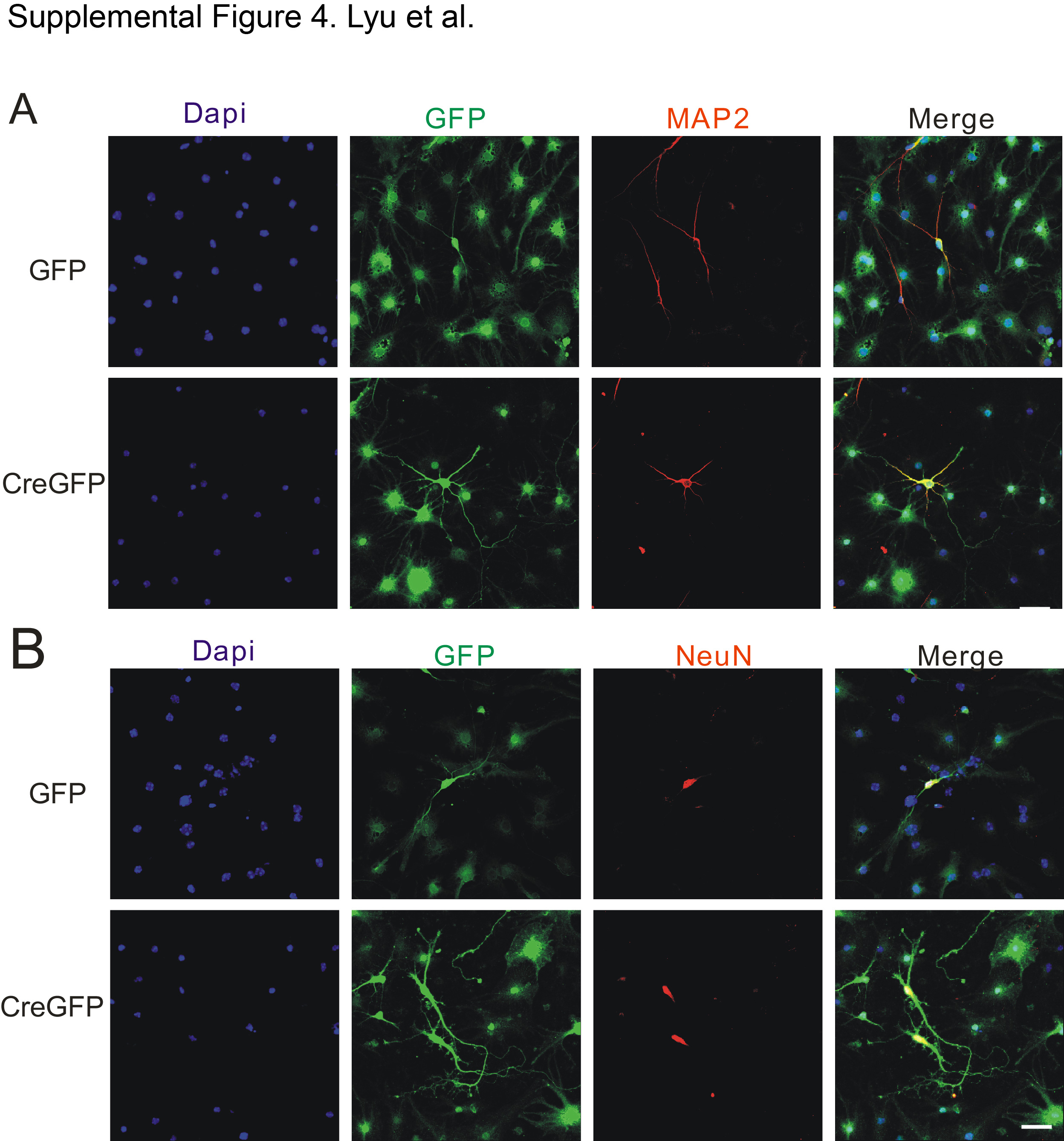

Supplement: Additional file 4: Figure S4. — Neurons, differentiated from NSCs, develop into mature neurons. (A-B) Cells differentiated from NSCs 10 days after GFP or CreGFP lentivirus transfectionwere labeled with mature neuronal markers MAP2 and NeuN. Scale bar = 40 μm. [file 13041_2015_114_MOESM4_ESM.jpeg]

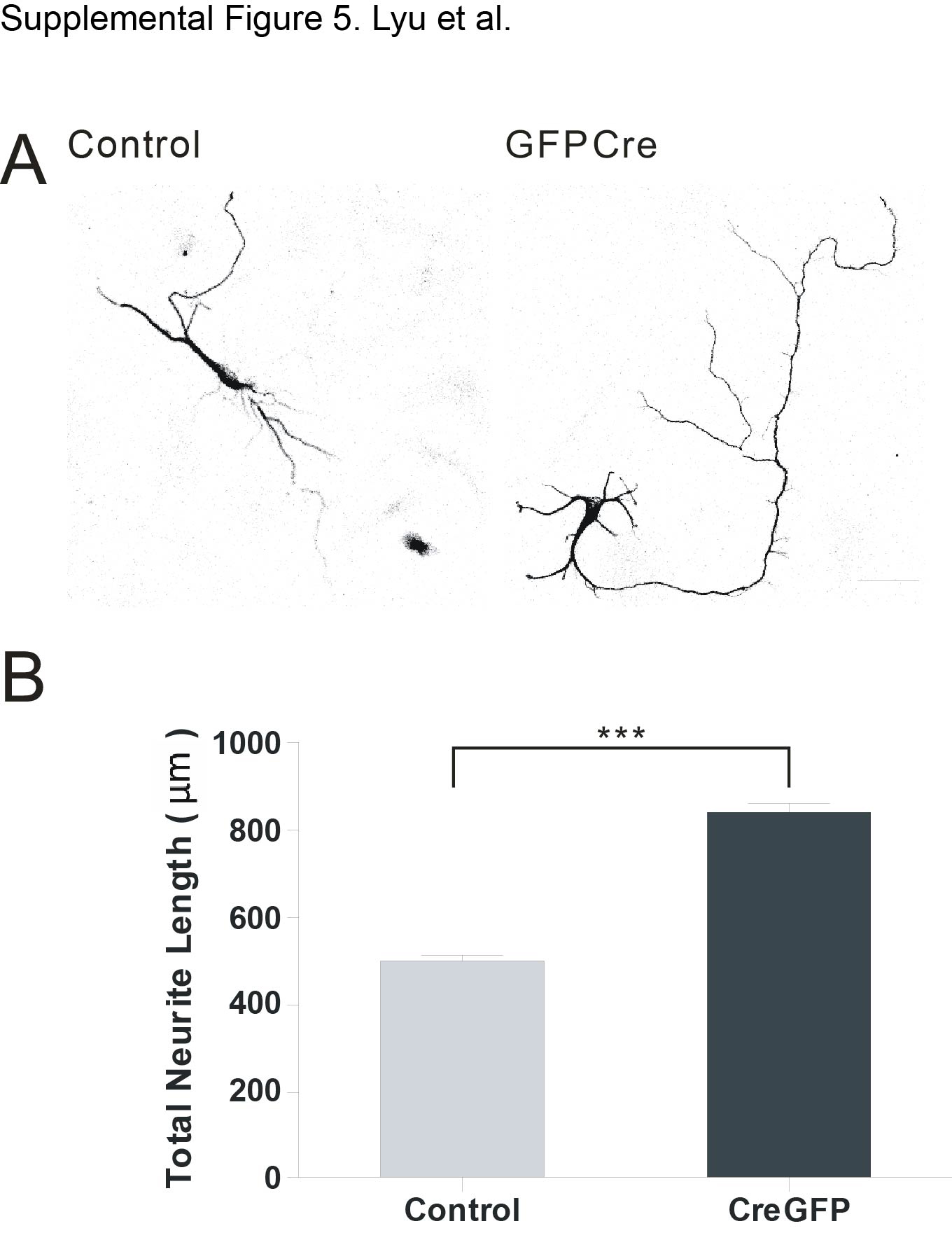

Supplement: Additional file 5: Figure S5. — Total neurite length of control and Pten −/− neurons. (A) Representative image of neurite growth of differentiated neurons at 5 days after transfectionwith GFP or CreGFP lentivirus. Scale bar = 40 μm (B) The quantification plot of neurite length of differentiated neurons. Transfection with lentivirus expressing CreGFP showed a remarkable increase in neurite length compared to control. [file 13041_2015_114_MOESM5_ESM.jpeg]
